# Ageing Analysis of Light-Emitting Diodes Used in Consumer Lighting †

**DOI:** 10.3390/ma19143134

**Published:** 2026-07-21

**Authors:** Levente Ákos Ludvig, Bianka Forczek, Gábor Harsányi

**Affiliations:** Department of Electronics Technology, Faculty of Electrical Engineering and Informatics, Budapest University of Technology and Economics, Műegyetem rkp. 3., H-1111 Budapest, Hungaryharsanyi.gabor@vik.bme.hu (G.H.)

**Keywords:** LED, ageing, phosphors, degradation, reliability, 2835 LED packages

## Abstract

This study investigates the degradation mechanisms of 2835-packaged white LEDs commonly used in residential lighting scenarios under various environmental conditions and explores how standard ageing tests compare to those that better reflect real-world use cases. The samples consist of commercially available LED strips, both cool and warm white. The luminous output of each LED on a strip is spectrally measured individually before ageing, then aged under particular conditions for a certain time and measured individually again. The measurements for a given LED are then analysed from multiple aspects, including material degradation processes during ageing, for example by following changes in phosphor efficiency.

## 1. Introduction

The aim of this research project is to assess the discrepancies between the manufacturer-specified and observed lifespans of residential LED-based lighting products, extending our earlier study presented at ISSE 2025 [1]. It is often found that such light sources become defective much sooner than the specifications would suggest [2]. This might be caused by the failure of the drive circuitry [3], but this is not the only possible cause. Certain reports also mention the failure of the LEDs themselves, sometimes also indicated by a black spot in the phosphor layer [4]. In other cases, the failure of the product might not be catastrophic; it is also reported that the luminous output of LED-based light sources tends to decay over their lifetime, as well as shift toward cooler correlated colour temperatures (CCTs) [5,6].

LED ageing affects both the quantity and quality of the light emitted. This study aims to quantitatively track these changes to understand the underlying degradation mechanisms by analysing variations in luminous flux, colour shift, and phosphor efficiency parameters. The scope of the project includes assessing how Peck’s lifetime model applies to the results obtained, as well as defining its limitations in this specific experiment.

The degradation of LED-based lighting solutions has been studied extensively in high-power devices, most importantly in automotive and industrial applications. Degradation is a multi-mechanism process involving changes at the die level, in the phosphor layer, and at the package level in the encapsulation. The contribution of the processes at these levels varies across devices and even individual samples, depending heavily on the operating conditions, most notably electrical drive current, temperature and humidity [7].

Operating at elevated temperatures has been shown to increase the degradation of the phosphor material and yellowing of the transparent encapsulant that embeds the phosphor particles, whereas humidity can be linked to the hydrolysis of phosphors and corrosion of metallic lead frames, interconnects and reflectors [8].

Among the materials in the package, the phosphor is the most susceptible to degradation. Studies indicate that this alone is responsible for 33% of the total luminous output loss, while the respective loss of the semiconductor diode is only 12% [9]. White LEDs are commonly made with YAG:Ce^3+^ phosphor, due to its high quantum efficiency and broad emission spectrum that peaks around 550 nm [10]. YAG:Ce^3+^ is, however, susceptible to moisture-induced hydrolysis, which has the effect of roughening particle surfaces and reducing conversion efficiency over time, a process that may be characterised using a two-stage activation energy model [11]. In warm white LED packages, phosphors are often combined with red-emitting nitride phosphors, such as SrAlSiN_3_:Eu^2+^, to lower the correlated colour temperature [12]. While these nitride compositions generally exhibit good thermal stability, their long-term behaviour remains less well documented.

The majority of published ageing studies focus on high-power LED packages, often driven at currents exceeding 350 mA, whereas the low-power packages prevalent in consumer products, such as the 2835 package often used on LED strips, receive comparatively little attention [13,14]. While standard testing and qualification protocols, most importantly the 85 °C/85% RH steady-state ageing test, are useful for benchmarking, they do not necessarily replicate the environmental conditions encountered in typical residential installations, such as high-humidity bathrooms and kitchens at near-ambient temperatures or enclosed luminaires with poor thermal management [15]. Due to this discrepancy, several authors have questioned the applicability of Arrhenius-based lifetime models that are commonly applied in ageing studies [9,16].

Arrhenius-based lifetime models are used to calculate the so-called acceleration factor (AF), which describes the ratio between the rate of degradation in the ageing environment and normal use. It is presumed that ageing tests accelerate the degradation of the devices. Peck’s lifetime model is one extension of these models, incorporating relative humidity alongside temperature into the calculation. The AF is given by the formula in Equation (2), considering the temperature and relative humidity levels of the environment, as well as the maximal operating values of these environmental conditions given by the manufacturer.

Beyond the phosphor layer, the protective and optical materials in LED packages also face significant stresses that compromise performance [4,7]:

1. Encapsulant degradation: Silicone or epoxy-based polymers embedding phosphor particles undergo photothermal stress from blue photon absorption and heat from the die. This leads to polymer chain scission, oxidation, and yellowing, which reduces light extraction efficiency and causes colour shifts. Micro-cracking can create pathways for moisture ingress, accelerating other degradation modes [17].
2. Reflector degradation: Silver-plated reflective surfaces in SMD packages can tarnish or corrode, particularly in humid environments or when exposed to sulphur compounds, reducing light extraction efficiency.
3. Lead frame and interconnects: Corrosion of lead frame materials or degradation of die attach materials increases thermal resistance, raising junction temperatures and accelerating temperature-dependent degradation. Thermo-mechanical stress from power cycling can cause solder joint fatigue [9,18].

## 2. Materials and Methods

To examine the degradation of the samples, ageing was accelerated by operating the LEDs in climatic chambers at predefined temperature and humidity levels. For the tests, the following environments were chosen:

1. Room temperature with high humidity, mimicking conditions that LEDs might be exposed to in kitchens and bathrooms: 25 ± 5 °C, 95 ± 3% RH (further cited as 25/95).
2. High-temperature dry air, simulating a scenario where the LEDs are installed with inadequate cooling, in which the absolute water content of the high-temperature air mimics that of conventional room-temperature air: 85 °C, 5 ± 3% RH (further cited as 85/05).
3. Standard 85/85 test—conditions often used in the industry to accelerate ageing of electronic devices (called steady-state thermal humidity test)—used as a reference: 85 °C, 85% RH (further cited as 85/85 and as reference chamber).

The 85/85 reference test was performed in an industrial climate chamber which, by design, applies strong forced-air circulation. The 25/95 and 85/05 environments were realised in separate chambers without forced convection; this contrast is deliberate, as these conditions are intended to reproduce realistic installation scenarios, such as enclosed luminaires and humid rooms, in which the LEDs are not exposed to forced airflow. In all chambers, the strips were placed on their sides on an open metal grid, which supported the samples from below while allowing air to circulate freely around them.

The samples comprised pieces of cool and warm white LED strips. The samples were placed into the chambers, and after every 500 h of ageing, a set of cool white and warm white samples was removed from the chambers. It is essential to note that no samples were returned to the chambers so that drying and reintroduction into a humid environment could not affect the results.

During ageing, the strips were driven from a laboratory power supply operating in constant-voltage mode at 12 V, placed outside the chambers. On the strips, groups of three LEDs are connected in series with a current-limiting resistor, resulting in an operating current of approximately 20 mA and a dissipated power of approximately 50 mW per LED package. The driving current remained stable throughout the ageing experiments.

Prior to ageing, every LED on each full strip was measured to establish individual baselines. The strips were then cut into segments, and one segment per colour and per condition was permanently removed from the chambers at each 500 h interval, so that every aged LED could be compared directly with its own pre-ageing measurement. The number of individually measured LEDs per condition and colour was 12 at 500 h, 9 at 1000 h and at 1500 h, and 6 at 2000 h; the experiment was originally planned for 1500 h and was extended during operation, which is reflected in the decreasing segment sizes. Data collected beyond 2000 h were excluded from the analysis due to the low remaining sample count. In the statistical analysis, the individual LED package was used as the statistical unit; it should be noted that the LEDs measured at a given time point originate from the same strip segment and are therefore not fully independent samples, which is a limitation of the present design. At the same time, the paired before/after design, in which every LED serves as its own baseline, controls for the considerable unit-to-unit variability of these devices and increases the statistical sensitivity of the comparisons; the consistency of the observed trends across conditions, colours and time points, together with the ANOVA and Tukey HSD results, supports the robustness of the reported tendencies.

**Figure 1:**
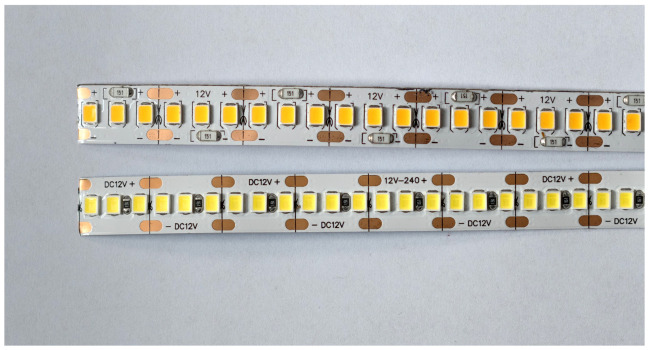
Photo of the warm white (above) and cool white (below) LED strips.

Each LED on each strip, as indicated in Figure 1, was measured before and after the ageing using a Konica Minolta CS-1000A spectroradiometer (Konica Minolta, Inc., Tokyo, Japan), and thus the spectral power distribution (SPD) was obtained for all samples before and after the degradation process.

**Figure 2:**
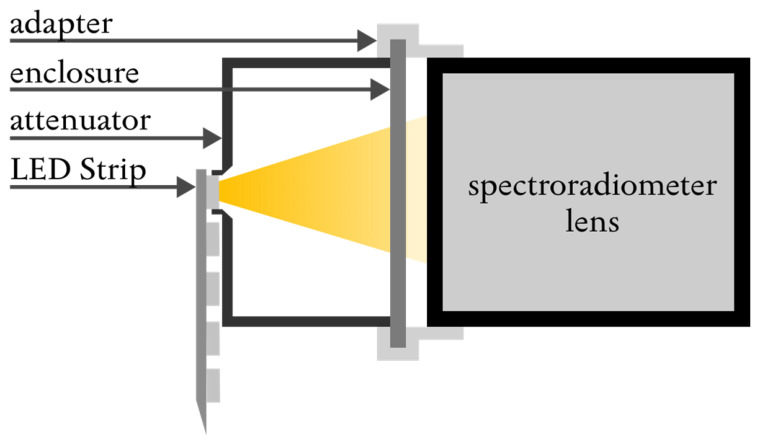
Schematic diagram of the measurement assembly.

To obtain more comprehensive data, the methodology was optimised to enable the measurement of individual LEDs. This approach allows for direct before/after comparisons of each LED unit, providing significantly higher-resolution data than traditional bulk measurement techniques. To achieve this, a custom adapter was designed and manufactured that allowed the sample strip to be attached to the measurement device. The LED to be measured was firmly held and precisely positioned to ensure the reliability and reproducibility of the results, whereas the light emitted by all other LEDs on the sample was obscured. To keep the irradiation within the measurement range of the spectroradiometer, an attenuator was used. The characteristics of this filter were determined independently and were corrected for. The schematic diagram of the measurement is shown in Figure 2.

**Figure 3:**
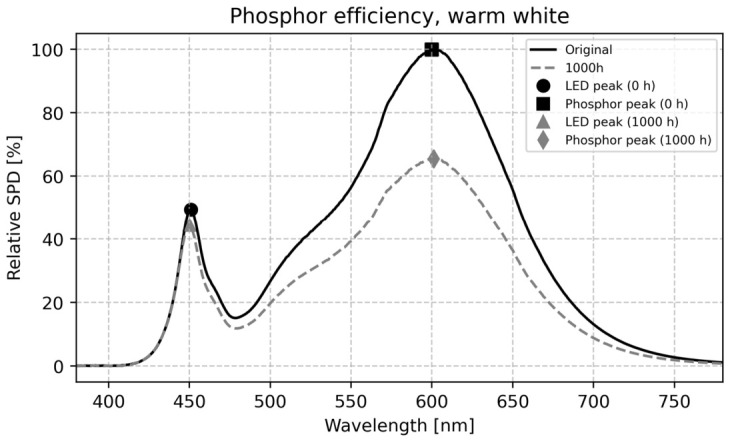
Visualisation of peaks for phosphor efficiency calculation for warm white LEDs.

**Figure 4:**
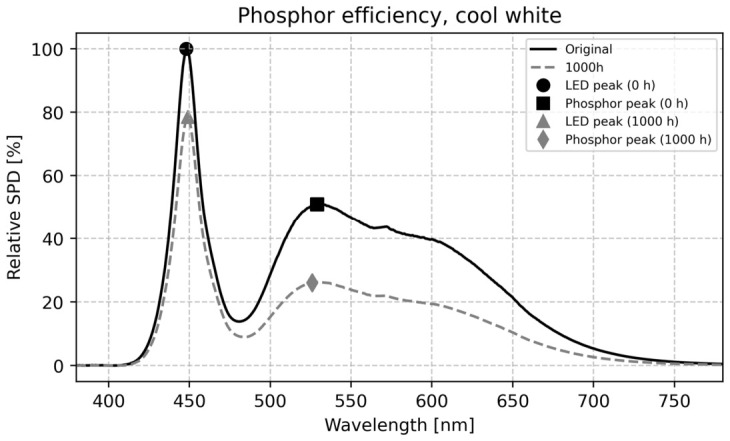
Visualisation of peaks for phosphor efficiency calculation for cool white LEDs.

From the SPDs, the following metrics were derived:

1. Relative luminous flux [%]: Providing insight into the loss of light from the end-user’s standpoint. This quantifies the overall light emission as a percentage of the initial luminous flux, directly reflecting the perceived brightness reduction over the lifespan of the LED.
2. Correlated colour temperature (CCT) [K]: To assess the shift towards cooler colours, which impairs usability. This is calculated using McCamy’s model [19]. McCamy’s approximation calculates the CCT from the CIE 1931 chromaticity coordinates (x, y) as CCT = −449n^3^ + 3525n^2^ − 6823.3n + 5520.33, where n = (x − 0.3320)/(y − 0.1858) [19]. As the LEDs age, the phosphor layer tends to degrade and contribute less towards the light output of the package. As the phosphor is responsible for providing the lower-energy portion of the SPD, this deterioration leads to cooler correlated colour temperatures. This colour shift not only affects the quality of the light aesthetically but might also impact functionality in applications where this is critical, such as in medical environments, museums or retail spaces. In residential scenarios, excessive amounts of blue light might also influence circadian rhythms, thus making shifts in correlated colour temperature a key aspect when analysing the degradation of illuminants.
3. Phosphor efficiency (relative) [%]: This is defined as the ratio of spectral power at the peak wavelength of the phosphor to that at the peak wavelength of the blue LED exciting it; see Figure 3 and Figure 4 for details. In both warm and cool white LEDs, the emission spectrum exhibits distinct peaks at around 450 nm and between 500 and 600 nm, corresponding to the blue chip and the phosphor, respectively. This ratio is a useful diagnostic indicator of the state of the phosphor. Should the ratio decrease gradually over the lifespan of the samples, it would suggest that the phosphor is losing its ability to convert blue light into lower-energy green/yellow/red light. Conversely, if the ratio remains constant, it suggests that the loss of luminous flux is caused by other factors, such as the degradation of the blue chip or the packaging losing its transparency. The applicability of this peak-based metric was verified by tracking the peak wavelengths themselves: the median blue-chip and phosphor peak positions remained stable within ±2 nm across all ageing conditions and time points, with no systematic drift. As the observed peak displacements are within the wavelength accuracy of the measurement and show no tendency, the peak ratio is not confounded by peak shift or spectral broadening and can be regarded as a reliable indicator of the phosphor conversion efficiency.

The phosphor efficiency calculation is indicated in Equation (1):

(1) $$\eta_{phosphor,t}=\frac{{SPD}_{t,\lambda =\lambda \left(phosphor\right)}}{{SPD}_{t,\lambda =\lambda (diode)}}$$

where:

- $\eta_{phosphor,t}$ is the phosphor efficiency [%];
- ${SPD}_{t,\lambda =\lambda \left(phosphor\right)}$ is SPD at phosphor peak [%];
- ${SPD}_{t,\lambda =\lambda (diode)}$ is SPD at diode (LED) peak [%];
- t is the time point (e.g., 500 h).

### 2.1. Peck Model

Using Peck’s model [20], the acceleration factor for the 85/85 test was calculated as given in Equation (2).

(2) $$AF={\left(\frac{RH_{t}}{RH_{u}}\right)}^{n}e^{\frac{E_{a}}{k}\left(\frac{1}{T_{u}}-\frac{1}{T_{t}}\right)}$$

where:

- RH is relative humidity [%];
- n is a constant (chosen as 2.66 based on Peck’s publication [20]);
- $E_{a}$ is the activation energy (assumed to be 0.5 [eV] [21,22,23]);
- k is Boltzmann’s constant (8.617333262 × 10^−5^ [eV/K]);
- T is temperature [K];
- u suffix stands for use, in this case the specified operating temperature/humidity (40 °C/60% RH);
- t suffix stands for test, i.e., the temperature/humidity the device was operated at in the chamber (85 °C/85% RH).

Thus, the acceleration factor in the 85/85 environment is calculated to be approximately AF = 25 according to the parameters measured and published previously in the literature [20,21,22,23].

The calculation for the 25/95 and 85/05 conditions yields approximately AF = 1.3 and AF = 0.01, respectively, meaning the model predicts barely any acceleration or even a deceleration in the simulated environments.

### 2.2. Junction Temperature Characterisation

The junction temperature elevation of the investigated LED type was characterised by thermal transient measurements (T3Ster/TeraLED, MicReD Ltd., Budapest, Hungary), on three samples per colour at the strip operating current, yielding a junction-to-reference thermal resistance of 120–142 K/W, i.e., an elevation of only 6–7 K at the dissipated power of approximately 50 mW when the package is externally cooled. Without forced cooling, infrared thermographic estimates for this package type indicate a junction temperature approximately 10–15 K above the ambient, whereas the forced convection of the 85/85 reference chamber keeps the packages close to the chamber temperature. This offset is an inherent part of the installation scenarios reproduced by the still-air chambers, and it is small compared with the 60 K difference between the ageing conditions.

## 3. Results

### 3.1. Optical Parameters

The following optical metrics were derived from the spectral measurements to characterise the degradation of the samples.

#### 3.1.1. Luminous Flux

**Figure 5:**
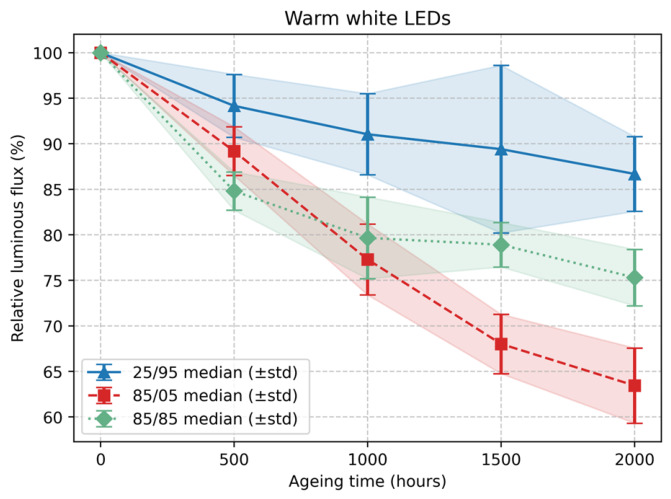
Degradation in relative luminous flux for warm white LEDs; datapoints represent the median value of samples for the given times and conditions, and error bars represent standard deviation.

**Figure 6:**
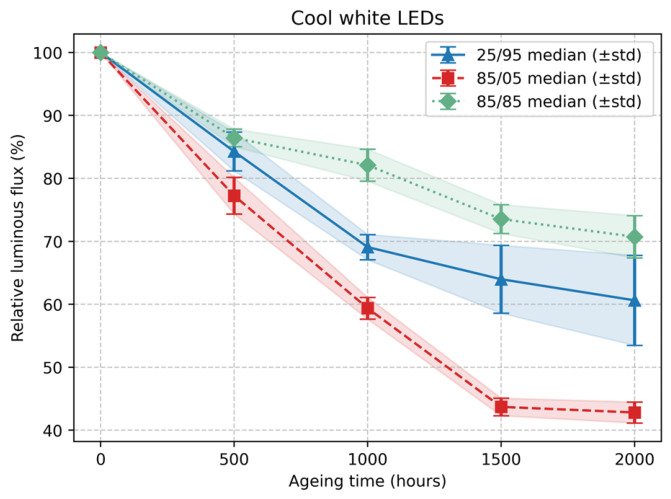
Degradation in relative luminous flux for cool white LEDs; datapoints represent the median value of samples for the given times and conditions, and error bars represent standard deviation.

Comparison of the luminous flux measurements in Figure 5 and Figure 6 indicates that cool white LEDs exhibit a faster luminous-flux decay than warm white LEDs.

It is apparent that the degradation was generally more rapid in the environmental conditions meant to mimic real-world scenarios than in the standard 85/85 chamber, especially in the case of warm white LEDs.

Whilst the warm white samples show a steady decay over 2000 h, the luminous flux levels of the cool white samples seem to level off after the first 1500 h, suggesting that the degradation mechanism responsible for part of the decay in luminous flux has slowed down significantly after that point, especially in the case of the 85/05 test.

Judging each graph’s evolution separately, the contrast between the 85/05 and 25/95 conditions is noteworthy: the curves in the 25/95 environment show a deceleration in the rate of ageing even after just 500 h, whereas in the case of the 85/05 chamber, the rate of ageing is closer to being constant (excluding the sharp change from 1500 h to 2000 h in the case of cool white samples). This suggests that the dominant degradation mechanisms under high-humidity and high-temperature conditions are fundamentally different. Moisture-driven processes, such as hydrolysis of the phosphor and encapsulant, are known to approach equilibrium once the available material surfaces are consumed and the encapsulant reaches moisture saturation [16,24], while thermally driven degradation alone proceeds continuously, as long as the elevated temperature is maintained.

**Figure 7:**
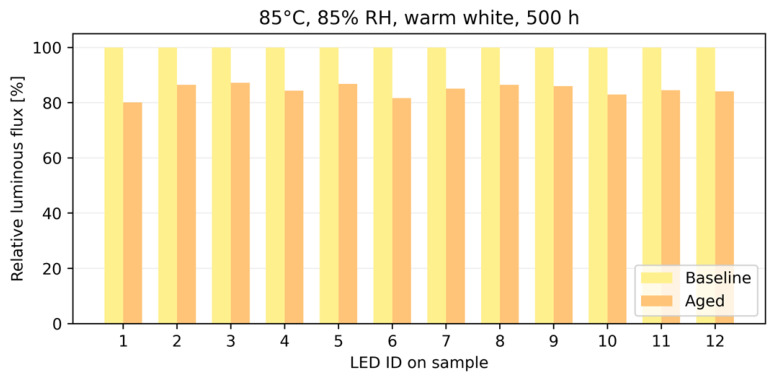
Relative luminous flux of individual warm white LEDs on a given sample aged in the 85/85 chamber for 500 h.

**Figure 8:**
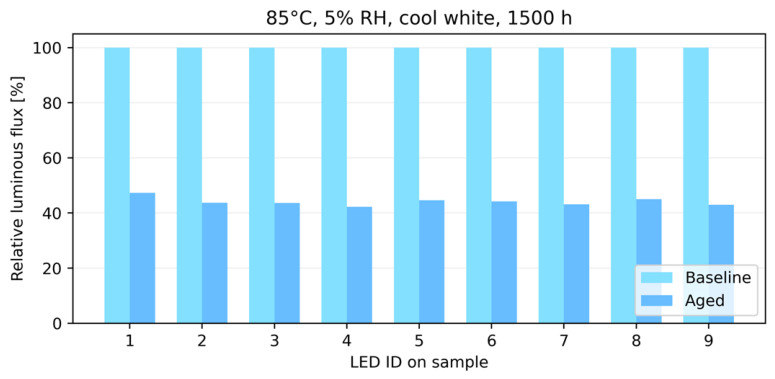
Relative luminous flux of individual cool white LEDs on a given sample aged in the 85/05 chamber for 1500 h.

**Figure 9:**
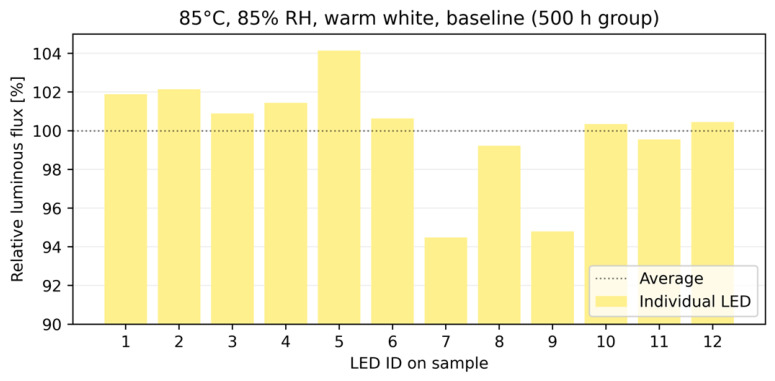
Relative luminous flux of individual warm white LEDs on the same sample prior to ageing (0 h), normalised to the sample average (100%), and corresponding to the LEDs analysed in Figure 7.

**Figure 10:**
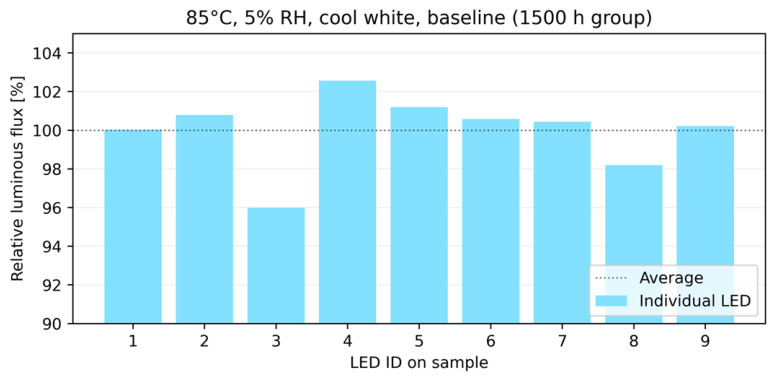
Relative luminous flux of individual cool white LEDs on the same sample prior to ageing (0 h), normalised to the sample average (100%), and corresponding to the LEDs analysed in Figure 8.

It is crucial to highlight that the individual packages on such LED strips show a great degree of variation regarding light output, colour temperature and SPD in general; therefore, it was of utmost importance to make comparisons between the before and after ageing stages on the same samples [13]. Analysis of the measured data confirms this heterogeneity in the ageing. The spread observed in the data, manifesting in high standard deviations for datapoints, is driven largely by the uneven ageing characteristics and general inhomogeneity of the LEDs on a given sample. The inhomogeneous ageing is demonstrated by two typical examples in Figure 7 and Figure 8, while Figure 9 and Figure 10 show the corresponding inhomogeneity in relative luminous flux for the same LEDs prior to ageing.

The relative luminous flux values for both warm and cool white LEDs for all the ageing conditions are listed in Table A1 and Table A2 in Appendix A.

#### 3.1.2. CCT

The correlated colour temperature shows a tendency towards higher values (i.e., cooler colours) throughout all experiments. The degradation in the quality of the light produced by the LEDs is more prominent in the case of the cool white samples. The samples in the 85/05 chamber have undergone the most substantial change; after 2000 h of ageing, the light was visibly blue [14,21].

**Figure 11:**
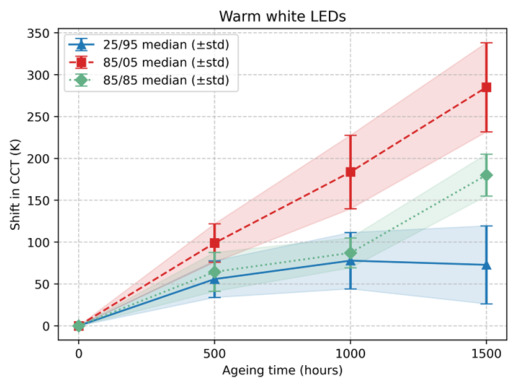
Shift in CCT; datapoints represent the median value of samples for warm white LEDs for the given times and conditions, and error bars represent standard deviation.

**Figure 12:**
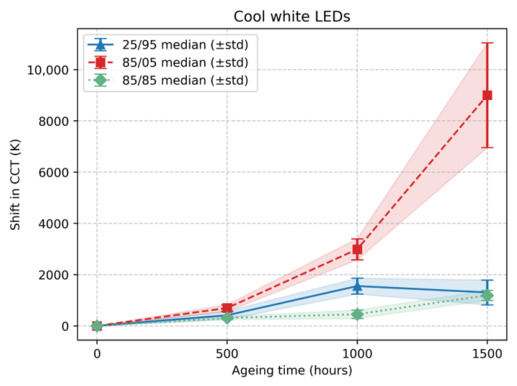
Shift in CCT; datapoints represent the median value of samples for cool white LEDs for the given times and conditions, and error bars represent standard deviation.

Due to some LEDs failing, the sample count for the 2000 h measurements proved insufficient for more advanced calculations; thus, the CCT and phosphor efficiency results could not be obtained for this time point. The CCT shifts of the warm and cool white LEDs are presented in Figure 11 and Figure 12, respectively.

It should be noted that McCamy’s approximation is optimised for near-white chromaticity coordinates and becomes increasingly unreliable as the spectral power distribution departs from white light. The CCT values exceeding several thousand Kelvin, as observed for the cool white samples aged in the 85/05 condition, should therefore be interpreted qualitatively rather than as precise colour temperatures; they indicate that the emitted light has shifted far from the white region of the chromaticity diagram.

The CCT shift values for both warm and cool white LEDs for all the ageing conditions are listed in Table A3 and Table A4 in Appendix A.

#### 3.1.3. Phosphor Efficiency

**Figure 13:**
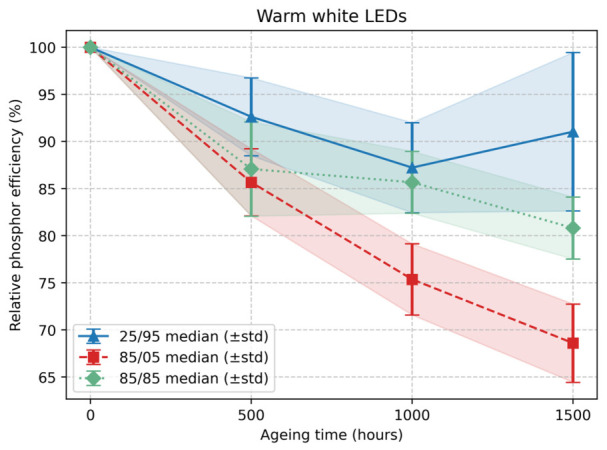
Degradation in relative phosphor efficiency; datapoints represent the median value of samples for warm white LEDs for the given times and conditions, and error bars represent standard deviation.

**Figure 14:**
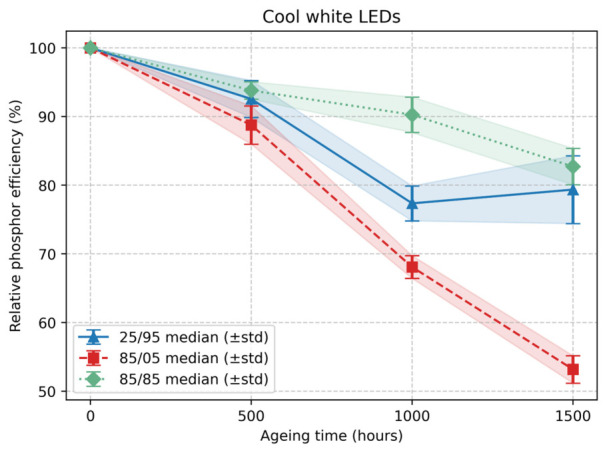
Degradation in relative phosphor efficiency; datapoints represent the median value of samples for cool white LEDs for the given times and conditions, and error bars represent standard deviation.

From the phosphor efficiency measurement, it is apparent that the degradation of the light-converting material, as shown in Figure 13 and Figure 14, is more prominent in the case of cool white samples. Similar to the relative luminous flux results, the rate of decay is more notable under simulated real-world conditions than in the 85/85 reference chamber [25].

#### 3.1.4. Spectral Analysis

**Figure 15:**
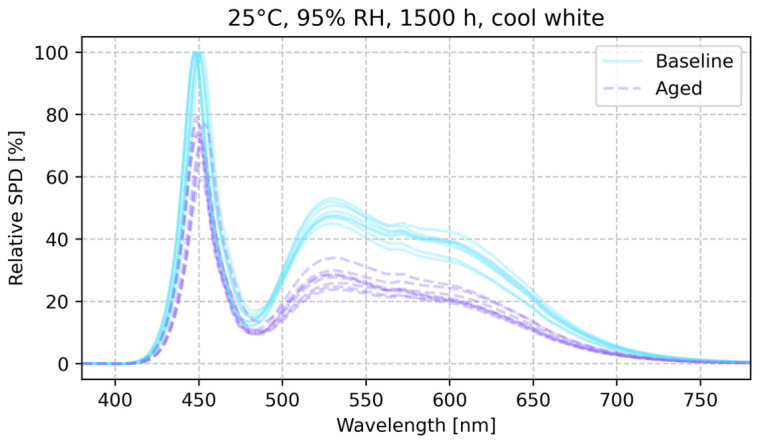
Comparison of baseline and aged spectra for the 25/95 environment after 1500 h of ageing for cool white LEDs.

**Figure 16:**
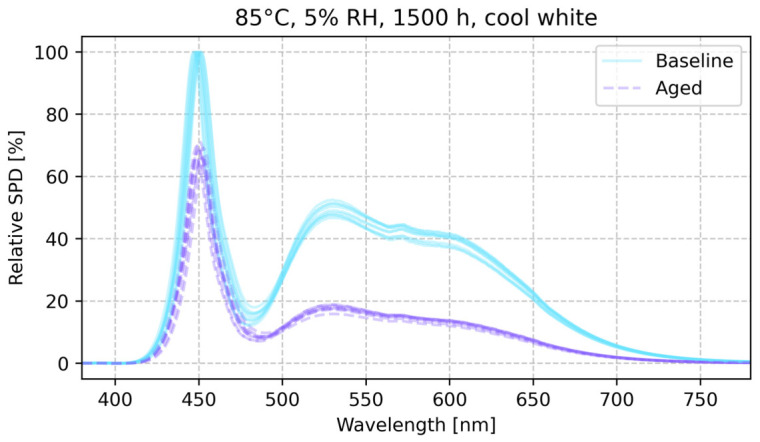
Comparison of baseline and aged spectra for the 85/05 environment after 1500 h of ageing for cool white LEDs.

A comparison of the original and degraded spectra in specific cases confirms the role of the phosphor and demonstrates both the changes in its contribution and the minimal extent to which the observed light loss can be attributed to the blue chip [21,22]. Figure 15 and Figure 16 compare the baseline and aged spectra of cool white LEDs after 1500 h in the 25/95 and 85/05 environments, respectively.

Analysing the spectra for the aforementioned environments, it is evident that the phosphor has undergone a much more conspicuous degradation in the 85/05 chamber compared to the 25/95, suggesting that both humidity and temperature cause significant ageing, but the effect of the latter is more pronounced on the phosphor, compared to its effect on the blue chip (the loss of light in this case can either be attributed to the degradation of the chip, or the packaging letting less light through) [17].

**Figure 17:**
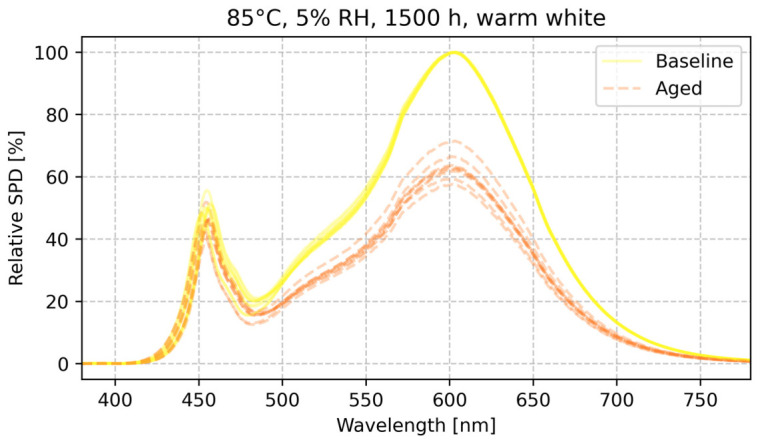
Comparison of baseline and aged spectra for the 85/05 environment after 1500 h of ageing for warm white LEDs.

In the case of warm white LEDs, the spectral data reveal a similar behaviour: as shown in Figure 17, the blue-chip emission remains essentially unchanged after 1500 h in the 85/05 environment, whereas the phosphor-converted portion of the spectrum exhibits a decrease in intensity. This further supports the conclusion that, under these ageing conditions, the phosphor is the component most susceptible to environmental stress and subsequent degradation.

The calculated phosphor efficiency values are listed in Table A5 and Table A6 in Appendix A for all three conditions.

**Figure 18:**
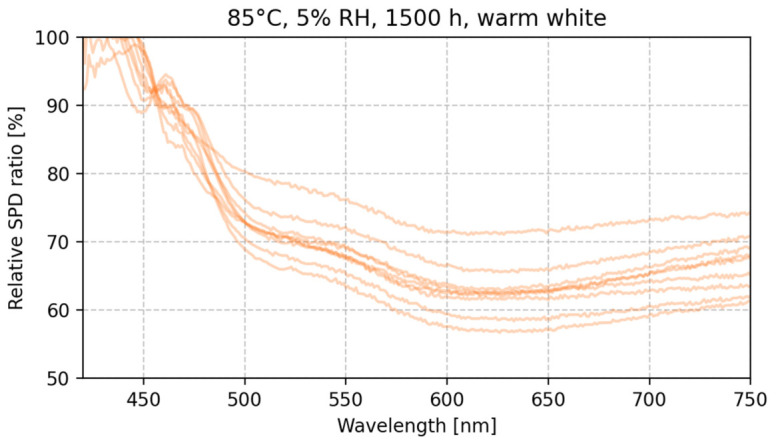
Relative spectral power distribution ratio versus wavelength. A value of 70% at 500 nm means that the luminous output at that wavelength has decreased by 30% during the ageing process.

Forming the quotient of the baseline/aged spectral power provides an intriguing insight into the phosphor used in the warm white LEDs, represented in Figure 18.

The resulting diagram visualises the relative spectral power and highlights that the phosphor has suffered a greater decrease between 600 and 650 nm than between 500 and 550 nm, suggesting that the phosphor layer is composed of a phosphor blend, the compounds of which degrade differently [24,26].

### 3.2. Material Analysis

To complement the optical characterisation, the phosphor composition and packaging condition of the samples were examined.

#### 3.2.1. Phosphor Material Analysis

SEM-EDS results and the spectral measurements provide information about the phosphor materials used for the measured 2835-packaged white LEDs. The SEM-EDS analysis was performed using an FEI Inspect S50 scanning electron microscope (FEI Company, Hillsboro, OR, USA), screening specifically for elements that are commonly found in white-LED phosphor compositions. The spectral measurements highlight the peak values corresponding to the phosphor material elements. The measured SEM-EDS atomic percentage is listed below in Table 1. The quantitative composition is reported with the customary accuracy of approximately 1 at. %; the actual accuracy depends on the degree of overlap between neighbouring peaks in the spectrum. Values below this accuracy, marked with an asterisk in Table 1, therefore indicate the detected presence of the corresponding element rather than a precise concentration. The presence of the activator elements Ce and Eu is nevertheless clearly established by their distinct, well-separated peaks in the measured spectra. Furthermore, SEM-EDS alone cannot conclusively identify phosphor phases, oxidation states or crystal structures; the phosphor assignments below are therefore considered consistent with, rather than proven by, the presented measurements, and complementary techniques such as photoluminescence spectroscopy or X-ray diffraction would be required for a definitive identification.

**Figure 19:**
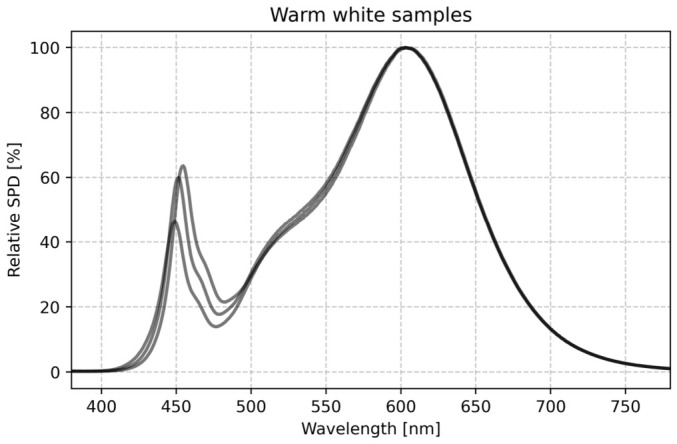
Relative spectral power distribution for 3 warm white samples.

Warm white LEDs exhibit peaks at around 540 nm and 610 nm (see Figure 19). One of the phosphor materials used is assumed to be Y_3_Al_5_O_12_:Ce^3+^, which is a commonly used phosphor for white LEDs, with the corresponding peak wavelength of 536 nm [26,27], as shown in Table 2. All the elements within Y_3_Al_5_O_12_:Ce^3+^ are detectable in the SEM-EDS spectrum. The 610 nm peak can likely be attributed to another phosphor, SrAlSiN_3_:Eu^2+^, given that its peak wavelength is listed as 610 nm and all the elements are detectable in the SEM-EDS spectrum; however, SrAlSiN_3_:Eu^2+^ is not as commonly used as the previously mentioned Y_3_Al_5_O_12_:Ce^3+^ phosphor [10,11]. The co-use of Y_3_Al_5_O_12_:Ce^3+^ and (Sr,Ca)AlSiN_3_:Eu^2+^ in a composite phosphor structure for warm white LEDs is also described in the literature, yielding CCTs comparable to the ones observed in our warm white samples [12].

**Figure 20:**
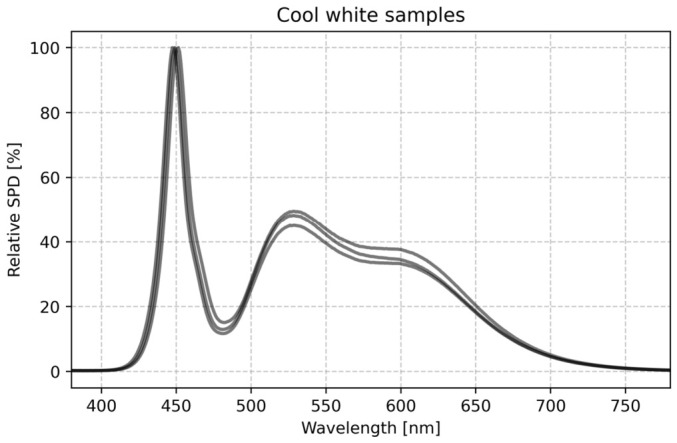
Relative spectral power distribution for 3 cool white samples.

Cool white LEDs exhibit peaks at around 520 nm, 540 nm and 610 nm, as shown in Figure 20. Based on the SEM-EDS results, the phosphors corresponding to the 540 nm and 610 nm peaks are presumably identical to those found in warm white samples. The 520 nm peak is only present in the case of the cool white LEDs and is likely emitted by BaYSi_4_N_7_:Eu^2+^ with the corresponding wavelength of 520 nm. Despite all the elements being present in the SEM-EDS spectrum, the evidence is not conclusive and shows a greater degree of uncertainty than previous findings.

The relative spectral power distribution for three warm and three cool white samples is shown below.

Table 2 lists the three phosphor materials with their corresponding emission wavelengths [26,28,29,30].

#### 3.2.2. Packaging Material Degradation

**Figure 21:**
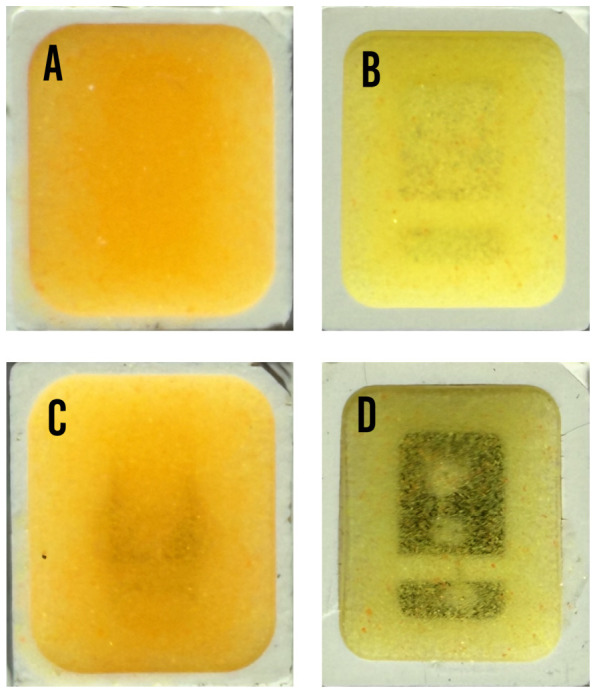
Warm white (**A**,**C**) and cool white (**B**,**D**) samples aged in condition (2) for 2000 h (**C**,**D**) compared to unaged ones (**A**,**B**).

Degradation of the packaging materials might also play a role in the loss of luminous flux, as it has an influence on light absorption and might also affect thermal characteristics of the operation [31,32]. Encapsulant materials are often silicone or epoxy-based and might lose their transparency over time. In our experiments, the transparent matrix material did not turn opaque; instead, its transparency increased during the ageing process, as seen in Figure 21. This suggests that the yellowing of the encapsulant itself did not play a critical role in the loss of luminous flux during the ageing process. However, visual inspection of the aged samples revealed corrosion of metallic surfaces, suggesting the degradation of the reflector could play a role in the loss of luminous flux by decreasing the light extraction efficiency. Additionally, corrosion of the lead frame and reflector materials can increase the thermal resistance of the package, raising the junction temperature and potentially accelerating other temperature-dependent degradation processes [4,33].

**Figure 22:**
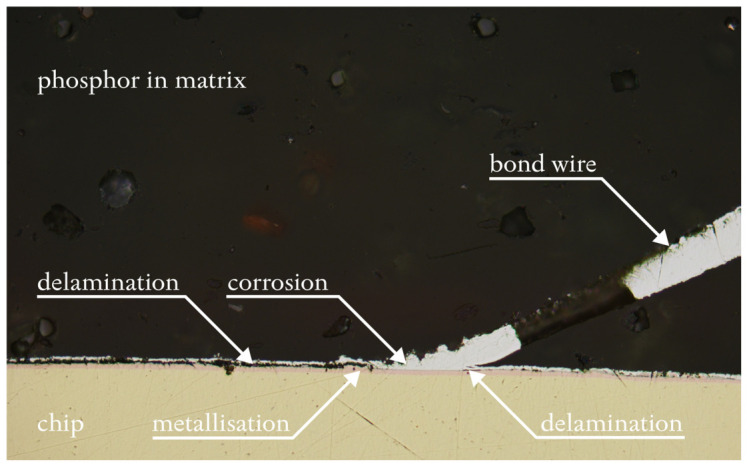
Cool white LED cross-section under the microscope after 1500 h of ageing at 25/95; the corrosion and delamination discussed are indicated by arrows.

While the opacity of the encapsulant influences luminous flux, other degradation mechanisms inside the package might result in different types of failures, such as intermittent operation or open-circuit behaviour. Damage to the bonding wire is of particular interest in this regard [4,7]. One of the cool-white samples began to exhibit flashing behaviour following the ageing process and was therefore selected for cross-sectional analysis, which revealed that the bondwire attachment to the chip was already in a suboptimal condition. This degradation is likely responsible for the flashing observed during post-ageing operation and could be caused by the mismatch of thermal expansion coefficients between the materials [33]. Furthermore, progressive degradation of the bond wire increases its series resistance, which can limit the current reaching the die and consequently reduce the luminous flux output. The observed corrosion and delamination are indicated in Figure 22.

## 4. Discussion

The results highlight that one of the most apparent effects of LED degradation is the loss of luminous flux, which is primarily attributed to the decay in the phosphor’s ability to convert part of the blue light to a wide-band reddish light. Consequently, the light appears bluer and dimmer to the human end-user.

Phosphor is known to be sensitive to both elevated temperatures and high humidity. Although the degradation mechanisms observed in the warm white samples appeared similar to those in the cool white samples, the rate of degradation was noticeably lower. This suggests that the phosphor blend used in the warm white samples is more resilient to the environmental conditions that the LEDs were exposed to during the experiments [27]. Furthermore, warm white LEDs typically contain a higher phosphor concentration, as indicated by the larger phosphor-to-blue peak ratio in their SPD [34]. Consequently, a given degree of phosphor degradation may have a less pronounced effect on the overall optical output compared to cool white samples, where the lower phosphor loading makes the impact more immediately apparent.

Based on the observations, the LEDs seem to degrade faster under the simulated real-world conditions (85/05 and 25/95) than in the reference chamber (85/85). The numerical data is represented in Table 3, Table 4 and Table 5. A contributing factor may be the strong forced convection present, by design, in the industrial environmental chamber used for the reference test, which improves heat removal from the packages. This convection was deliberately absent in the other two chambers, as the simulated environments are intended to reproduce realistic installation scenarios in which no forced airflow is present. As discussed in Section 2.2, the junction temperature in the still-air chambers is estimated to run approximately 10–15 K above the ambient, whereas the forced convection of the reference chamber keeps the packages close to the chamber temperature; this thermal difference is an inherent part of the reproduced installation scenarios. The comparison therefore reflects the combined effect of the environmental conditions and the realistic thermal boundary conditions of the intended use cases, rather than differences in self-heating. This highlights the discrepancy between the standard testing procedure and experiments closer to real-world use cases, and suggests that in some cases, the standard test might favour the LEDs rather than accelerating degradation [2,9,15,25,35].

One-way ANOVA confirmed that the ageing condition has a statistically significant effect on all three metrics at every measured time point (*p* < 0.05). Tukey HSD post hoc comparisons reveal that, while 85/05 remains significantly different from both other conditions at all time points (q > 5 in all cases), the difference between 25/95 and 85/85 loses significance for warm white luminous flux at 1000 h (q = 3.11) and 1500 h (q = 2.77), as well as for warm white phosphor efficiency at 1000 h (q = 1.67) and 1500 h (q = 2.56). This supports the interpretation that humidity-driven degradation approaches a saturation point, while thermally dominated degradation continues progressively.

Based on the AF calculated using Peck’s model, the LEDs would be expected to age approximately 25 times faster in the case of the 85/85 ageing chamber than in normal operation [20]. In this environment, warm and cool white LEDs reached a relative luminous flux output of 80% at roughly 1000 and 1200 h, respectively. Given the AF, this would mean that the samples should last 25,000–30,000 h in normal operation, which is rarely observed for any commercially available lighting product. Whilst the model’s results are roughly correct for this environment, this model cannot be applied to the other two chambers, where the test temperature or humidity falls short of the manufacturer specifications, resulting in the acceleration factors of 1.3 and 0.01 calculated in Section 2, meaning the model predicts barely any acceleration or even a deceleration. This contradicts the experimental findings of this paper, as in both simulated conditions the degradation was at least as significant as in the reference chamber.

It is noteworthy that Peck’s model was originally developed for modelling the ageing of packaged integrated circuits and electronic modules, where the dominant failure mechanisms are more uniform. While the model is capable of making predictions in certain ageing scenarios where both temperature and humidity exceed the specified operating levels, its applicability to LEDs is limited, as LED degradation involves a wider range of mechanisms, such as phosphor degradation, encapsulant yellowing, and reflector corrosion, that are not present in the original scope of the model [20].

The most obvious sign of LED degradation is the decline in luminous performance, which results from the combined effects of various stressors alongside temperature, for example, hygromechanical stress (a combination of humidity and mechanical stress factors). Electronic stressors, such as current fluctuations or voltage spikes, can also accelerate LED degradation [18]. This is further supported by the observation that degradation kinetics differ significantly between the 25/95 and 85/05 tests. Hydrolysis of phosphor/silicone composites has been shown to approach equilibrium as the reaction progresses [24]. The activation energy for hydrolysis itself decreases with moisture content, meaning the degradation rate diminishes as the system saturates [16]. This is consistent with the levelling-off observed in the 25/95 samples and highlights a fundamental limitation of the Peck model, which assumes a constant activation energy throughout the degradation process. Beyond this, while considering environmental temperature, the model also neglects the thermal processes occurring within the LED itself, further limiting its predictive accuracy. To improve lifetime predictions for LEDs, refinement of the existing model or the adoption of alternative approaches is needed. It is noteworthy that the projection method widely used in the lighting industry, IES TM-21, which extrapolates lumen-maintenance data measured according to LM-80, does not incorporate humidity at all [36], underlining the absence of an established humidity-aware lifetime model for LEDs. More general multi-stress formulations, such as the Eyring family of models from which Peck’s model derives [37], or two-stage activation energy descriptions of moisture-driven degradation [11,16], offer possible directions. Based on the present results, a multi-mechanism approach combining a saturating, humidity-driven term with a progressive, thermally driven term would describe the observed behaviour more faithfully than any single-mechanism acceleration model.

## Figures and Tables

**Table 1:** SEM-EDS results.

Element	Atomic [%] Warm White LEDs	Atomic [%] Cool White LEDs
C	47.03	40.61
N	0.63 *	-
O	20.93	28.38
Na	0.09 *	-
Mg	0.03 *	-
Al	9.15	12.05
Si	9.43	1.65
S	0.04 *	-
Cl	0.06 *	0.16 *
K	0.11 *	0.10 *
Ca	0.28 *	-
Ti	0.15 *	1.83
Cr	-	0.09 *
Fe	-	0.29 *
Ni	-	0.03 *
Cu	0.02 *	0.12 *
Ga	3.30	0.01 *
Sr	1.97	5.47
Y	6.64	3.50
Zr	-	0.07 *
Ba	-	5.42
Ce	0.12 *	0.03 *
Eu	0.03 *	0.20 *

* Below the approximately 1 at. % quantification accuracy; the value indicates the detected presence of the element rather than a precise concentration.

**Table 2:** Listed phosphor materials with their corresponding wavelength.

Phosphor Material	Emission Wavelength (nm)
Y_3_Al_5_O_12_:Ce^3+^	536
SrAlSiN_3_:Eu^2+^	610
BaYSi_4_N_7_:Eu^2+^	520

**Table 3:** Median luminous flux loss (%) relative to baseline (0 h).

	Warm White			Cool White		
Ageing Time (h)	25/95	85/05	85/85	25/95	85/05	85/85
500	5.8	10.8	15.2	15.7	22.7	13.6
1000	9.0	22.7	20.3	30.9	40.6	17.9
1500	10.6	32.0	21.1	36.0	56.3	26.5
2000	13.3	36.6	24.7	39.4	57.2	29.3

**Table 4:** Median phosphor efficiency loss (%) relative to baseline (0 h).

	Warm White			Cool White		
Ageing Time (h)	25/95	85/05	85/85	25/95	85/05	85/85
500	7.4	14.3	12.9	7.4	11.2	6.2
1000	12.8	24.6	14.3	22.7	31.9	9.7
1500	9.0	31.4	19.2	20.7	46.8	17.3

**Table 5:** Median CCT shift (K) relative to baseline (0 h).

	Warm White			Cool White		
Ageing Time (h)	25/95	85/05	85/85	25/95	85/05	85/85
500	56	99	64	415	698	308
1000	78	184	87	1554	2989	454
1500	73	285	180	1306	9003	1192

## Data Availability

The original contributions presented in this study are included in the article. Further inquiries can be directed to the corresponding author.

## Abbreviations

The following abbreviations are used in this manuscript:

LED	Light-Emitting Diode
CCT	Correlated Colour Temperature
RH	Relative Humidity
SPD	Spectral Power Distribution
AF	Acceleration Factor
SEM	Scanning Electron Microscopy
SEM-EDS	Scanning Electron Microscopy with Energy-Dispersive Spectroscopy

## Appendix A

The measured median values and standard deviations of the relative luminous flux, the CCT shift and the relative phosphor efficiency are listed for all ageing conditions in Table A1, Table A2, Table A3, Table A4, Table A5 and Table A6.

**Table A1:** Relative luminous flux for warm white LEDs: medians ± standard deviations.

Ageing Time (h)	25/95 Condition	85/05 Condition	85/85 Condition
0	100.00 ± 0.00	100.00 ± 0.00	100.00 ± 0.00
500	94.16 ± 3.46	89.19 ± 2.67	84.81 ± 2.10
1000	91.04 ± 4.45	77.29 ± 3.90	79.66 ± 4.49
1500	89.41 ± 9.20	68.01 ± 3.26	78.91 ± 2.44
2000	86.68 ± 4.11	63.45 ± 4.13	75.30 ± 3.10

**Table A2:** Relative luminous flux for cool white LEDs: medians ± standard deviations.

Ageing Time (h)	25/95 Condition	85/05 Condition	85/85 Condition
0	100.00 ± 0.00	100.00 ± 0.00	100.00 ± 0.00
500	84.27 ± 3.08	77.26 ± 2.91	86.43 ± 1.40
1000	69.08 ± 2.01	59.37 ± 1.73	82.09 ± 2.54
1500	63.97 ± 5.39	43.70 ± 1.39	73.53 ± 2.29
2000	60.62 ± 7.15	42.81 ± 1.66	70.72 ± 3.36

**Table A3:** CCT shift for warm white LEDs: medians ± standard deviations.

Ageing Time (h)	25/95 Condition	85/05 Condition	85/85 Condition
0	0 ± 0	0 ± 0	0 ± 0
500	56 ± 22	99 ± 23	64 ± 23
1000	78 ± 34	184 ± 44	87 ± 18
1500	73 ± 47	285 ± 53	180 ± 25

**Table A4:** CCT shift for cool white LEDs: medians ± standard deviations.

Ageing Time (h)	25/95 Condition	85/05 Condition	85/85 Condition
0	0 ± 0	0 ± 0	0 ± 0
500	415 ± 159	698 ± 134	308 ± 61
1000	1554 ± 308	2989 ± 410	454 ± 164
1500	1306 ± 485	9003 ± 2047	1192 ± 193

**Table A5:** Phosphor efficiency for warm white LEDs: medians ± standard deviations.

Ageing Time (h)	25/95 Condition	85/05 Condition	85/85 Condition
0	100.00 ± 0.00	100.00 ± 0.00	100.00 ± 0.00
500	92.62 ± 4.14	85.67 ± 3.56	87.07 ± 5.00
1000	87.22 ± 4.78	75.37 ± 3.77	85.67 ± 3.29
1500	91.03 ± 8.40	68.61 ± 4.15	80.81 ± 3.29

**Table A6:** Phosphor efficiency for cool white LEDs: medians ± standard deviations.

Ageing Time (h)	25/95 Condition	85/05 Condition	85/85 Condition
0	100.00 ± 0.00	100.00 ± 0.00	100.00 ± 0.00
500	92.55 ± 2.70	88.76 ± 2.79	93.79 ± 1.26
1000	77.35 ± 2.54	68.08 ± 1.65	90.25 ± 2.58
1500	79.35 ± 4.93	53.17 ± 1.99	82.71 ± 2.65
